# Odours in Asphalt: Analysis of the Release of H_2_S from Bitumen by a Mass Spectrometric Residual Gas Analyser

**DOI:** 10.3390/mps7040055

**Published:** 2024-07-15

**Authors:** Marcello Monteleone, Giuseppe Di Luca, Marcello Filomia, Alessio Fuoco, Alberto Figoli, Johannes Carolus Jansen

**Affiliations:** 1 Institute on Membrane Technology, National Research Council of Italy (CNR-ITM), via P. Bucci 17/C, 87036 Rende, Italy; g.diluca@itm.cnr.it (G.D.L.); a.fuoco@itm.cnr.it (A.F.); a.figoli@itm.cnr.it (A.F.); jc.jansen@itm.cnr.it (J.C.J.); 2 Department of Chemistry & Chemical Technologies, University of Calabria, via P. Bucci, 87036 Rende, Italy; 3 HA Italia S.p.A., Viale della Scienza, 78-80, 36100 Vicenza, Italy; marcello.filomia@ha-group.com

**Keywords:** bitumen, hydrogen sulphide evolution, mass spectrometry, residual gas analyser

## Abstract

During the production and laying phases of hot-mixing asphalt (HMA), various volatile organic compounds (VOCs) and noxious gases such as H_2_S are released into the atmosphere. These emissions are a serious environmental problem, a risk to human health, and expose workers and residents to unfriendly odours. The aim of this study was the development of a fast and sensitive analytical method to detect the H_2_S emitted from hot bituminous binder that is generally used in the various stages of asphalt production, processing, handling and during road construction. The method consisted in the analysis of evolved H_2_S from a flask with molten bitumen, using nitrogen as a carrier gas to lead the volatile compounds into a residual gas analyser equipped with a quadrupole mass spectrometer. The analysis was performed following the H_2_S-specific signals at *m*/*z* 33 (HS^+^) and at *m*/*z* 34 (H_2_S^+^) in real time, directly on the sample without laborious and expensive pre-treatments and with short response times (<6 s). Calibration with a standard mixture of 1000 ppm of H_2_S in nitrogen allows semi-quantitative H_2_S detection. The sensitivity and rapidity of the method were evaluated by quenching the release of sulphur compounds with commercial odour-suppressing agents. Upon addition of 0.1% of additive in two minutes, the H_2_S signal drops about 80% in two minutes, confirming the good response of the method, even with a very complex matrix.

## 1. Introduction

Bitumen is an important petroleum derivative that is widely used for the production of sealants, waterproofing and flooring [1,2], with an annual production of about 100 million tons [1,3]. Market demand is growing, and it is estimated that it will increase by about 4% within the next 5 years [4]. Hot-mixing asphalt (HMA) production takes place at temperatures between 150 °C and 180 °C, and the temperature increases significantly for plants that recycle hot-milled asphalt [5,6]. The HMA is transported in suitably covered trucks to the manufacturing site, where it is compacted at a temperature of 140–160 °C [7]. During the storage and handling of the bitumen at these temperatures, a complex mixture of predominantly hydrocarbons is released into the atmosphere.

In recent years, population growth and the expansion of urban centres are the reason that the sources of odorous industrial emissions are closer to inhabited centres [8,9]. In this context, there is growing attention for the emission of HMA-related pollutants to the atmosphere, and in particular, the odorous emissions produced in HMA production plants are of great concern. Emissions related to HMA production and processing are temperature-dependent and depend on a dynamic equilibrium of gases, vapours, aerosols, combustion products (CO, CO_2_, NO_x_ and SO_x_), organic compounds of various species, including volatile organic compounds (VOCs), polycyclic aromatic hydrocarbons (PAHs), hydrogen sulphide (H_2_S) and others [10]. Among these, VOCs and NO_x_ contribute to the formation of a series of secondary compounds, such as peroxyacetyl nitrate, aldehydes, acids and tropospheric ozone, that may be noxious to plants [11] or have adverse short- and long-term health effects [11,12]. H_2_S is a colourless, flammable compound associated with a rotten egg smell, with a very low perception threshold (<1 ppm). It is very toxic, and exposure to concentrations above 1000 ppm is deadly [13]. The minimum risk level inhalation of H_2_S recommended by the Agency for Toxic Substances and Disease Registry (ATSDR) is 0.02 ppm. Exposure to concentrations above 500–1000 ppm can cause serious health damage in humans. Therefore, the Occupational Safety and Health Administration (OSHA) has determined the daily exposure limit in the general industry at 20 ppm. The EPA (U.S. Environmental Protection Agency) recommends an inhalation reference concentration (RFC) of 0.001 mg/m^3^ [14]. Bitumen fume concentrations can reach up to 30,000 ppm [15], exposing operators and citizens to serious health risks.

Various regulations aimed at containing emissions of HMA production plants have been drawn up for dust, particulate matter (PM_10_, PM_2.5_) and greenhouse gases (CO_2_, NO_x_, SO_2_). However, no specific limits have been set for the odorous compounds [16,17]. The scientific literature has extensively reported the emissions from HMA in terms of airborne substances and toxicological risk, linked above all to occupational exposure, but odours were not considered [18,19]. Bad odours from industrial plants are increasingly considered air pollutants and can have a significant negative impact on both quality of life and economic activity [6]. The odorous emissions emitted during the various stages of production, movement and installation of HMA mixtures severely limit the usability of the area and are an indisputable and persistent cause of annoyance for the resident population, for passersby and insiders [5]. Odour emissions are therefore one of the main concerns for the hot-mix asphalt industry, the EAPA (European Asphalt Pavement Association) in Europe and the NAPA (National Asphalt Pavement Association) in the United States, which have started technical discussions to address this strategic issue [11].

In recent years, olfactometry, measurement of environmental odour, has been standardized internationally only for certain types of applications (waste, wastewater treatment and composting plants, landfills) [20,21]. The regulation of odour is complex and depends on various subjective factors. There is no international regulation framework, but different approaches are used, sometimes also at the local/regional level [22]. Therefore, it is necessary to develop procedures and analytical methods to identify and quantify the molecules responsible for the smell in HMA production [16]. Currently, only parameter emissions such as particulate matter, total hydrocarbons and polycyclic aromatics are controlled in bitumen production plants [23].

Several well-established analytical techniques have been developed to characterise VOCs, such as thermogravimetric mass spectrometry (TG-MS), gas chromatography–mass spectrometry (GC–MS) and liquid chromatography–mass spectrometry (LC–MS) [10,24,25]. These analyses are of fundamental importance for identification and quantification of the pollutants released into the atmosphere, but they do not provide any information on the odour perception produced by the mixture as a whole [23]. Correlation between odour perception and chemical–physical properties of odorous molecules has not yet been clarified, and research is underway, especially for complex gaseous mixtures such as those generated by bitumen [26]. Sensor technology and electronics have enabled the development of devices capable of measuring and recognizing homogeneous mixtures characterized by single or complex compounds such as artificial olfactory systems (AOSs), also called electronic noses [27]. Some odour emission models from bitumen have been studied by means of an electronic nose, but no olfactometric data are available [23]. Only one study has been reported in the literature on odorous emissions due to H_2_S and its derivatives from asphalt plants [15]. Among the methodologies reported for petroleum products, none is specific to bitumen, and they all tend to suffer from problems related to matrix effects and interference with other compounds [28,29,30,31,32]. Despite technological advances, it will be difficult for electronic devices to have the same perception of the biological nose for bad smells [27]. In the analytical field, sample preparation is a crucial step of the entire procedure, especially in the case of complex matrices. When many steps are necessary, numerous drawbacks comprise a high consumption of organic solvents, increasing costs, loss of analyte, et cetera. Therefore, the way forward is to try to develop analytical methods that are simpler and faster [33]. Several methods are reported in the literature for the analysis of H_2_S in various matrices. An overview of the methods and their limitations is given in Table 1. 

In the analytical field, sample preparation is a crucial step of the entire procedure, especially in the case of complex matrices. When many steps are necessary, numerous drawbacks comprise a high consumption of organic solvents, increasing costs, loss of analyte, et cetera. Therefore, the way forward is to try to develop analytical methods that are simpler and faster [33]. In this work, we propose a versatile analytical method for real-time quantitative monitoring of the evolution of hydrogen sulphide and its derivatives from molten bitumen, using a carrier gas to lead the evolved gases and vapours from the headspace to the mass spectrometer. The proposed method allows real-time analysis of multiple gases (in this case, focused on H_2_S) with a high measurement frequency, with the possibility of providing important information on time-dependent phenomena which help in understanding even complex processes. The high specificity that mass spectrometry provides is furthermore a crucial aspect in the field of kinetic phenomena, where the developed method shows better performance in terms of response times compared to, for instance, electrochemical sensors in continuous analysis. In this work, the response times are <6 s. With sensors, on the other hand, response times in the range between 7 and 500 s are obtained [37]. With other routine methods such as gas chromatography (GC) or liquid chromatography (LC), on the other hand, it is not possible to perform real-time analysis, and often adequate sample preparation is required before analysis, which is not necessary with this method where the analysis is performed directly on the sample as it is, without further pre-treatment steps. This may lead to a more accurate odour control, where H_2_S blocking additives are quantitatively added on the basis of the amount of H_2_S present.

## 2. Experimental Section

### Materials

A road bitumen used as a bituminous binder with 70/100 dmm penetration, from the Alma Petroli S.p.A. refinery (Ravenna, Italy), was studied in this work. Its properties are reported in Table 2. Nitrogen, purity (99.99+%), used as a carrier gas and as an internal standard, and Argon BIP^®^ purity (99.9999+%), used as an inert gas to allow determination of the background signals, were purchased from SAPIO (Monza, Italy). A standard mixture of H_2_S/N_2_ (1000 ppm of H_2_S) was supplied by Risam Gas S.r.l (Milano, Italy).

Paraffin oil was used as a diluent and viscosity modifier, and was supplied by Sigma-Aldrich (Milano, Italy), CAS No. 8012-95-1.

HA.REMOVAL was used as received from HA Italia S.p.A (Vicenza, Italy).

## 3. Procedures

### 3.1. Setup for the H_2_S Evolution Experiments

**Figure 1:**
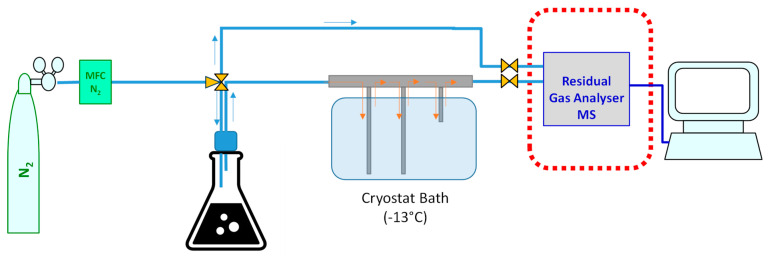
Experimental setup for sampling and analysis of H_2_S released from hot bitumen.

The H_2_S evolution was tested by a dynamic headspace analysis. A scheme of the experimental setup is shown in Figure 1. A bitumen sample (60 g) was molten in an oven at 150 °C and transferred into an Erlenmeyer flask equipped with a gas inlet and outlet, placed on a hot plate with magnetic stirring. Then, 10 g of paraffin oil was added to reduce the viscosity [41], and the mixture was kept under gentle stirring (800 rpm) at 150 °C, the temperature that is generally used in the asphalt production process [5,6]. The temperature was kept constant at 150 °C to guarantee similar conditions as those during the asphalt production process, and in order to perform the analysis as much as possible in the same conditions. The flask was maintained under continuous flux of nitrogen carrier gas (25 mL·min^−1^) to transfer the volatile compounds released from the sample into the analyser. The stirring speed and temperature were maintained constant to guarantee a stable release of the volatile compounds from the sample into the headspace.

#### 3.1.1. Residual Gas Analysis

Quantitative analysis of the evolved sulphur compounds (in the form of H_2_S) was performed with a mass spectrometric residual gas analyser device equipped with a quadrupole mass filter (HPR-20 QIC Benchtop residual gas analysis system for max. 200 AMU, Hiden Analytical, Warrington, UK). This setup was developed for the online analysis of light gases during membrane permeation and has the advantage of continuous monitoring of any known gas or vapour with high sensitivity and quick response time [42,43]. The flow rate of the carrier was controlled by EL-FLOW electronic Mass Flow Controllers (Bronkhorst High-Tech, Ruurlo, the Netherlands). The H_2_S was monitored by recording the characteristic signals at *m*/*z* = 34 amu (H_2_S^+^) and *m*/*z* = 33 amu (HS^+^), while the nitrogen was detected by *m*/*z* = 14 (N^+^), both using the secondary electron multiplier (SEM) detector. Nitrogen was monitored by following the weaker signal at *m*/*z* =14 (N^+^) and not the most abundant signal at *m*/*z* = 28 (N_2_^+^), in order to stay within the range of the SEM detector, which has an upper detection limit of 10^−6^ torr, above which it is necessary to use the Faraday detector. A standard certified H_2_S/N_2_ mixture with 1000 ppm of H_2_S was used for calibration. The sensitivity factor (RS) was calculated from the ratio of the signal (*I_i_*) of H_2_S and N_2_ and the ratio of the related flow rate (*Q_i_*), as described previously [42]:

(1) $$RS=\frac{I_{H_{2}S}}{I_{N_{2}}}\cdot \frac{Q_{N_{2}}}{Q_{H_{2}S}}$$

#### 3.1.2. H_2_S Evolution Experiments

Before analysis, the VOC-rich gas stream was passed through a cryogenic trapping system at −13 °C to remove the condensable compounds with high molecular weight to prevent them from reaching the mass spectrometer. The background signals (*I_BG,i_*) were measured on the pure nitrogen stream before each test, bypassing the sample flask with a series of three-way valves. These signals were subtracted from the raw signals (*I_raw,i_*) in order to obtain the effective values released from the sample.

(2) $$I_{i}=I_{raw,i}-I_{BG,i}$$

The unknown flow rate of the H_2_S released from the sample can be calculated from the ratio of the N_2_ signal with a known fixed flow rate and the measured H_2_S signal, using the previously determined RS value (Equation (1)) obtained by calibration of the system with the H_2_S-containing standard mixture. Considering that the volume of the headspace in the Erlenmeyer flask is around 100 mL and the flux of the carrier gas is 25 mL·min^−1^, the volume of gas in the headspace is replaced on average every 4 min, while the residence time of the sampled gas in the instrument before reaching the mass spectrometric gas analyser is approximately 20 s [42]. The signals are recorded at a much higher frequency, at regular intervals of about 6 s.

## 4. Results

### 4.1. Calibration

**Figure 2:**
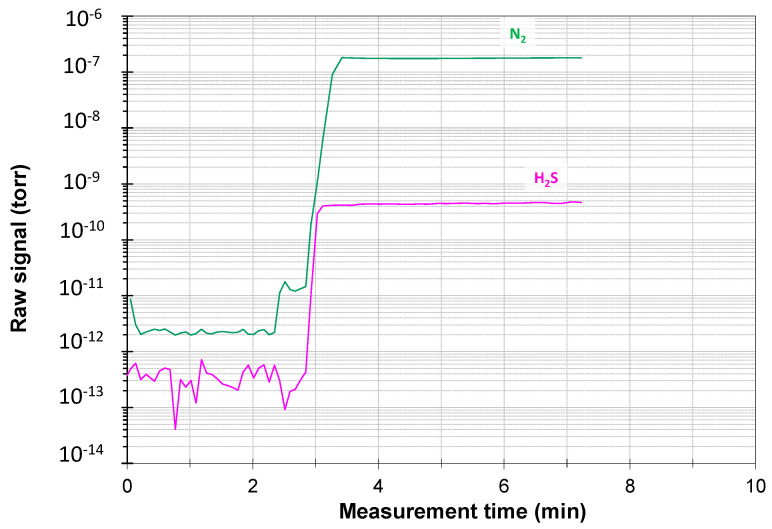
Raw unfiltered signal of argon for analysis of the background levels of N_2_ and H_2_S for about 2 min, followed by a standard mixture of 1000 ppm H_2_S in N_2_, showing the rapid response and high sensitivity after the step change from argon to the N_2_/H_2_S calibration mixture.

Preliminary tests were performed with a certified standard mixture of H_2_S (1000 ppm) in N_2_. This is a good reference value, considering the usual levels of H_2_S in asphalt [44]. Since 1 ppm of H_2_S in the liquid phase of asphalt corresponds to approximately 400 ppm in the vapour phase, asphalt with extremally high levels of H_2_S can reach 30,000 ppm of H_2_S in the vapour phase, and thus 1000 ppm of H_2_S is a representative concentration for a calibration standard. Tests with this standard mixture, fed directly into the gas analyser, allowed us to evaluate the response of the analysis system itself in well-defined conditions without other variables. Before this test, the background signals were measured while sampling an inert gas (argon), and the gas was then switched instantaneously to the calibration mixture. The raw signals of the test are shown in Figure 2, which demonstrates the rapid response of the analytical system to variations in concentrations. After the first ca. 3 min in which the background signals of the system are analysed, as soon as the standard mixture is sampled, both the N_2_ carrier signal and the H_2_S signal increase immediately, stabilizing in less than a minute. These two values are then used as a reference in data processing for the quantitative analysis of the unknown concentration in the H_2_S evolution experiments.

### 4.2. H_2_S Release and Detection

**Figure 3:**
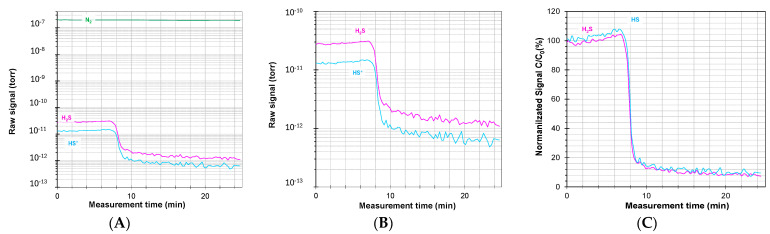
(**A**) Raw Mass Spectrometer signal with N_2_ carrier gas signal and (**B**) zoom excluding the carrier gas signal. (**C**) Normalized signals of H_2_S^+^ and HS^+^ with respect to their starting concentration, (C_H2S_^+^)/(C_H2S_^+^)_0_ (%) and (C_HS_^+^)/(C_HS_^+^)_0_ (%).

The bitumen was heated to 150 °C in the presence of paraffin oil prior to analysis of H_2_S. For the specific bituminous binder used in the present study, 10 g of paraffin oil was found to be the minimum quantity necessary for a sufficient reduction in the viscosity [41] to ensure continuous and stable magnetic stirring, which in turn is needed to guarantee a stable release of the volatile compounds from the sample into the headspace. Figure 3A,B report the raw signals as a function of time (with and without the N_2_ internal standard’s signal) of the H_2_S released from a bitumen sample analysed under the conditions described in Section 3.1.2. At the beginning of the sampling, the signals of H_2_S^+^ and HS^+^ are both stable in the range of 10^−10^–10^−11^ torr, demonstrating that both the flux of the carrier gas and the emission from the bitumen are stable. The N_2_ signal at *m*/*z* = 14 (N^+^ signal) is stable at 2 × 10^−7^ torr over the entire interval (Figure 3A). This is also an indication that the concentration of H_2_S in the headspace reached an equilibrium and is stable. Thus, all the subsequent variations in the signals are due to external factors. After 7 min, an aliquot of approximately 0.1 wt% of the HA.REMOVAL, relative to the amount of bitumen, was added with a syringe to the stirred hot bituminous sample. Almost immediately, there is a drastic drop of the specific signals of H_2_S: i.e., H_2_S^+^ and HS^+^. In fact, HA.REMOVAL is an additive commonly used as a scavenger of H_2_S from asphalt, and it is able to reduce the emission of hydrogen sulphide from bitumen even when added at low concentrations [45]. In addition to demonstrating its effectiveness as an H_2_S scavenger, this test demonstrates the fast response of the developed setup and method to follow the variation in the H_2_S concentration in the headspace of the hot bitumen flask: within about two minutes, half the time to refresh the headspace volume, the characteristic signals drop to less than 20% of their starting value. The two monitored signals have an identical profile, and both can be used for analytical purposes. In particular, the most abundant signal H_2_S^+^ (*m*/*z* = 34) can best be used for the quantification of the H_2_S emission, while the less abundant HS^+^ (*m*/*z* = 33) is useful for the unambiguous identification of the species, excluding possible overlap with other signals. This improves the specificity of the method for this application, avoiding interference with other compounds that may be present and can cause problems with the analysis, especially in the case of complex matrices such as asphalt. On the other hand, the S^+^ signal at *m*/*z* = 32 is unsuitable due to overlap with the O_2_^+^ signal, which may be present as a background signal or as a result of other oxygen-containing species and humidity in the sample. It must be noted that we follow the H_2_S^+^ and HS^+^ signals as markers for the H_2_S concentration in the headspace, but especially for HS^+^, we cannot exclude that this is also formed by fragmentation of volatile mercaptans that might be present in the headspace. However, the contribution of mercaptans is expected to be very low, first of all because most of them are condensed in the cold trap and secondly because the HS^+^ is one of the weakest signals of mercaptans. In any case, the H_2_S^+^/HS^+^ signal ratio of the H_2_S standard can be taken as a reference to check the presence of other sources of H_2_S^+^ and/or HS^+^ signals.

As shown above, an important feature of this method is that it can monitor rapid changes in multiple gas species, in this case to evaluate the capacity of additives to reduce the emission of H_2_S. For instance, the herein used additive HA.REMOVAL decreased both monitored signals to less than 10% in approximately 4 min after its addition, i.e., within one complete refreshment of the headspace by the carrier gas (Figure 3C). The comparison and the good agreement between the calculated reduction based on both the H_2_S^+^ and HS^+^ signals confirmed that there are no potential errors due to the presence of isobaric ions coming from other analytes. The analysis in real time and the possibility of acquiring data at a high frequency in the order of seconds is a powerful tool for in-depth study of transient phenomena which are very useful, for example, in kinetics studies and to measure the time that should elapse from the addition of the additive to the exposure of the workers to the emission. In this experiment, the instrumental response time is <6 s, and it could be reduced another order of magnitude, optimizing the specific settings of the mass spectrometer. Thus, these results open the possibility of using this setup for the development of analysis methods for other classes of volatile compounds such as mercaptans, exploiting the high sensitivity and specificity of mass spectrometry.

## 5. Conclusions

In the present work, a method for the detection of H_2_S released from a molten bitumen was developed, employing a mass-spectrometric residual gas analyser. The method is very sensitive to changes in the concentration of the analytes, in both time and quantity. The mass spectrometric gas analyser allows for the unambiguous identification of the analytes with high specificity even in the case of a complex sample matrix. This is because different signals can be recorded for fragments of the same molecule, creating a kind of fingerprint of the samples. Although it is recommended to condense less volatile species in a cold trap to avoid excessive contamination of the mass spectrometer, the method allows for the direct analysis of the gas released from the original sample without particular treatments in the sample preparation. This is extremely useful since it avoids for instance slow and laborious extraction and separation processes, and the use of organic solvents etc., thus reducing both the costs and the necessary time for the analysis. Moreover, the very quick response of the instrument offers the possibility to study the kinetics and/or the diffusion and emission of volatile compounds from other types of matrices, enabling the monitoring of the efficiency of additives for lowering the emissions, not only on the basis of absolute quantities but also in terms of the time needed to reach the minimum quantity. The highly flexible method offers perspectives for the analysis of H_2_S release from other sources as well, such as wastewater treatment plants, or for the analysis of other compounds with a sufficiently specific signal in mass spectrometry. The possibility to selectively monitor any other gas species extends its applicability to a wide variety of other application fields.

## Figures and Tables

**Table 1:** Representative examples of methods for H_2_S detection.

Method	Applications	Range	Limitations	Ref.
ASTM D 5705 (Colorimetric Test)	Standard Test Method for Measurement of Hydrogen Sulphide in the Vapor Phase Above Residual Fuel Oils	5–4000 ppm	Low repeatability, non-quantitative	[34]
ASTM D 4952 (Colorimetric Test)	Standard Test Method for Qualitative Analysis for Active Sulphur Species in Fuels and Solvents	-	Qualitative analysis, use of heavy metals	[34]
ASTM 6021 multiple headspace extraction procedure (MHE)	Standard Test Method for Measurement of Total Hydrogen Sulphide in Residual Fuels by Multiple Headspace Extraction and Sulphur-Specific Detection	0.01–100 ppm	High costs, indirect measurement, expert operator, complex computations	[34]
ASTM 5623 (GC method)	Standard Test Method for Sulphur Compounds in Light Petroleum Liquids by Gas Chromatography and Sulphur Selective Detection	0.1–100 ppm	High cost, limited analysable samples	[34]
Fluorescent probes	Aqueous samples, biological fluids.	probe-dependent	Selectivity, rate dependent on detection chemistry. Signal accumulation measurements difficult to quantify.	[35]
Methylene blue (MB)	Aqueous samples, biological fluids.	∼2 μM	Limited detection range extracts acid-labile sulphide potential, ROS bleaching does not provide real-time measurements.	[35]
Monobromobimane (mbb)	Aqueous samples, biological fluids.	∼2 nM	Sample preparation and storage require severe requirements for expensive reagents that do not provide real-time measurements.	[35]
Electrode	Aqueous samples, biological fluids.	5–300 nM	Frequent calibration is necessary. Sensitive to reproducibility of solution components between devices. Electrode lifetime.	[35]
GC method	Gaseous samples	nM range	Method not simple, multi-stage protocol from preparation to sampling.	[36]
Chemical sensor	Gaseous samples	Depending on the type of sensor	Limited selectivity (interfering gases). Dependence on relative humidity and changing environmental conditions.	[32]

**Table 2:** Chemical and physical properties of bitumen 70/100 dmm (Bitumen Binder, Alma Petroli S.p.A).

Composition		Physical Properties ^#^	
Element	Content (wt.%)	Property	Value (Unit)
Carbon	79–88	Penetration at 25 °C ^a^	70–100 (dmm) ^§^
Hydrogen	7–13	Softening point ^b^	46–54 (°C)
Sulphur	5–7.5	Viscosity at 135 °C ^c^	min 230 (mm^2^ s^−1^)
Oxygen	8		
Nitrogen	3		

^#^ These properties are evaluated according to the standard methods: ^a^ EN 1426 [38]; ^b^ EN 1427 [39] and ^c^ EN 12595 [40]. ^§^ 1 dmm = 0.1 mm = 10^−4^ m.

## Data Availability

The original contributions presented in the study are included in the article.
